# Supplementary material for: The Protective Effects of Sour Orange (Citrus aurantium L.) Polymethoxyflavones on Mice Irradiation-Induced Intestinal Injury
Source: Molecules. 2022 Mar 16;27(6):1934. doi: 10.3390/molecules27061934 (PMC8948989; doi:10.3390/molecules27061934)
Supplement: Supplementary file 1 [file molecules-27-01934-s001.zip › molecules-1625294-supplementary/3-Supplemental Materials/Table S1.pdf]

Table S1. GO/KEGG enrichment results of differential expressed genes obtained from GSE126507

ONTOLOGY	ID	Description	Gene Ratio	*p*. adjust	Gene IDs	Count	Z score
BP	GO:0051673	membrane disruption in other organism	12/736	2.78E-05	Defa17/Defa2/Defa21/Defa22/Defa23/Defa24/Defa3/Defa30/Defa31/Defa34/Defa5/Ltf	12	3.464102
BP	GO:0035456	response to interferon-beta	13/736	3.75E-05	F830016B08Rik/Gm12185/Gm4951/Ifi204/Ifi207/Ifi211/Ifi213/Ifitm3/Iigp1/Irgm1/Tgtp1/Tgtp2/Xaf1	13	3.605551
BP	GO:0019731	antibacterial humoral response	14/736	9.48E-05	Defa17/Defa2/Defa21/Defa22/Defa23/Defa24/Defa3/Defa30/Defa31/Defa34/Defa5/Ltf/Pla2g6/Spon2	14	3.207135
BP	GO:0035458	cellular response to interferon-beta	11/736	0.000139	F830016B08Rik/Gm12185/Gm4951/Ifi204/Ifi207/Ifi211/Ifi213/Iigp1/Irgm1/Tgtp1/Tgtp2	11	3.316625
BP	GO:0002227	innate immune response in mucosa	12/736	0.000139	Defa17/Defa2/Defa21/Defa22/Defa23/Defa24/Defa3/Defa30/Defa31/Defa34/Defa5/Ltf	12	3.464102
BP	GO:0050829	defense response to Gram-negative bacterium	16/736	0.000269	Cxcl5/Defa17/Defa2/Defa21/Defa22/Defa23/Defa24/Defa3/Defa30/Defa31/Defa34/Defa5/Iigp1/Il23a/Ltf/Lyz1	16	4
BP	GO:0002385	mucosal immune response	12/736	0.001259	Defa17/Defa2/Defa21/Defa22/Defa23/Defa24/Defa3/Defa30/Defa31/Defa34/Defa5/Ltf	12	3.464102
BP	GO:0002251	organ or tissue specific immune response	12/736	0.001501	Defa17/Defa2/Defa21/Defa22/Defa23/Defa24/Defa3/Defa30/Defa31/Defa34/Defa5/Ltf	12	3.464102
BP	GO:0019730	antimicrobial humoral response	15/736	0.001903	Cxcl5/Defa17/Defa2/Defa21/Defa22/Defa23/Defa24/Defa3/Defa30/Defa31/Defa34/Defa5/Ltf/Pla2g6/Spon2	15	3.356586
BP	GO:0061844	antimicrobial humoral immune response mediated by antimicrobial peptide	13/736	0.003952	Cxcl5/Defa17/Defa2/Defa21/Defa22/Defa23/Defa24/Defa3/Defa30/Defa31/Defa34/Defa5/Ltf	13	3.605551
BP	GO:0034404	nucleobase-containing small molecule biosynthetic process	18/736	0.004002	Adk/Aprt/Art2b/Cad/Ctps2/Dera/Enpp5/Entpd5/Gck/Hk1/Ifng/Igf1/Nme4/Nudt1/P2rx7/Pde4d/Pfkfb2/Prkag2	18	0.471405
BP	GO:0051271	negative regulation of cellular component movement	26/736	0.004002	Abhd6/Abr/Adora3/Adtrp/Angpt4/Ccl12/Cd300a/Cdkn1b/Dcn/Dusp10/Dusp22/Dusp3/Epha1/Il1rn/Il33/Kcne3/Krit1/Mctp1/Meox2/Myocd/Pbld1/Sema3f/Sema4a/Sema4c/Sema6c/Stard13	26	1.961161
BP	GO:0071222	cellular response to lipopolysaccharide	24/736	0.004196	Ccl12/Cxcl5/Defa17/Defa2/Defa21/Defa22/Defa23/Defa24/Defa3/Defa30/Defa31/Defa34/Defa5/Gbp10/Ifng/Il1rn/Ltf/Nlrp3/Nos2/Nr1h2/Pde4d/Plscr2/Sirpa/Spon2	24	3.265986

BP	GO:0032496	response to lipopolysaccharide	28/736	0.004196	Abr/Ccl12/Cxcl5/Defa17/Defa2/Defa21/Defa22/Defa23/Defa24/Defa3/Defa30/Defa31/Defa34/Defa5/Dusp10/Gbp10/Ifng/Il1rn/Ltf/Nlrp3/Nos2/Nr1h2/P2rx7/Pde4d/Plscr2/Ptgfr/Sirpa/Spon2	28	3.40168
BP	GO:0046578	regulation of Ras protein signal transduction	21/736	0.004196	Abr/Adra1b/Arfgef1/Arhgap24/Arhgef3/Arhgef39/Arpp19/Cyth2/Git1/Gpr35/Igf1/Iqsec2/Itsn2/Kalrn/Mfn2/Ntrk1/Plekhg2/Plekhg5/Rasgef1a/Spata13/Syngap1	21	-0.654654
BP	GO:0050830	defense response to Gram-positive bacterium	15/736	0.004434	Defa17/Defa2/Defa21/Defa22/Defa23/Defa24/Defa3/Defa30/Defa31/Defa34/Defa5/Gbp10/Lyz1/Nlrp3/P2rx7	15	3.356586
BP	GO:0071219	cellular response to molecule of bacterial origin	24/736	0.005569	Ccl12/Cxcl5/Defa17/Defa2/Defa21/Defa22/Defa23/Defa24/Defa3/Defa30/Defa31/Defa34/Defa5/Gbp10/Ifng/Il1rn/Ltf/Nlrp3/Nos2/Nr1h2/Pde4d/Plscr2/Sirpa/Spon2	24	3.265986
BP	GO:0048511	rhythmic process	24/736	0.005569	Arntl/Ciart/Cipc/Cry2/Dbp/Fbxl21/Hlf/Igf1/Mycbp2/Nfkb2/Nos2/Npas2/Nr1d2/Ntrk1/Per1/Per2/Per3/Pgr/Rorc/Sgpl1/Stat5a/Tef/Timeless/Usp2	24	0.816497
BP	GO:0071216	cellular response to biotic stimulus	25/736	0.006141	Bid/Ccl12/Cxcl5/Defa17/Defa2/Defa21/Defa22/Defa23/Defa24/Defa3/Defa30/Defa31/Defa34/Defa5/Gbp10/Ifng/Il1rn/Ltf/Nlrp3/Nos2/Nr1h2/Pde4d/Plscr2/Sirpa/Spon2	25	3
BP	GO:0046890	regulation of lipid biosynthetic process	17/736	0.006885	Abhd6/Acadvl/Adgrf5/Cyp17a1/Dhcr7/Gip/Htr2b/Ifng/Igf1/Nr1h2/Ormdl2/Pde8b/Pdk4/Pla2g6/Por/Prkcd/Slc27a1	17	1.212678
BP	GO:0002237	response to molecule of bacterial origin	28/736	0.006885	Abr/Ccl12/Cxcl5/Defa17/Defa2/Defa21/Defa22/Defa23/Defa24/Defa3/Defa30/Defa31/Defa34/Defa5/Dusp10/Gbp10/Ifng/Il1rn/Ltf/Nlrp3/Nos2/Nr1h2/P2rx7/Pde4d/Plscr2/Ptgfr/Sirpa/Spon2	28	3.40168
BP	GO:0019216	regulation of lipid metabolic process	26/736	0.006885	Abhd6/Acadvl/Adgrf5/Apobec1/Cyp17a1/Dhcr7/Fgfr3/Gip/Htr2b/Ifng/Igf1/Mtmr2/Nr1d2/Nr1h2/Nucb2/Ormdl2/Pde8b/Pdk4/Pla2g6/Por/Prkag2/Prkcd/Rorc/Slc27a1/Stat5a/Thrb	26	1.568929
BP	GO:0001662	behavioral fear response	9/736	0.006885	Eif4g1/Fbxl20/Gja1/Gng7/Kalrn/Lypd1/Morc1/Npas2/Pde8b	9	1
BP	GO:0048871	multicellular organismal	33/736	0.007418	Abhd6/Acadvl/Adgrf5/Ampd2/Ampd3/Arntl/Bmp8a/	33	-0.522233

		homeostasis			Cckar/Col2a1/Coro1a/Cytl1/Dram2/Ehmt1/Eif4g1/Gigyf2/Gja1/Gnas/Il18r1/Il1rn/Irx3/Ldb2/Mfn2/Muc13/Nox4/Nr1d2/Nr1h2/P2rx7/Pbld1/Pdk4/Per2/Ppp1r13l/Slc27a1/Slc28a2		
BP	GO:0002209	behavioral defense response	9/736	0.007418	Eif4g1/Fbxl20/Gja1/Gng7/Kalrn/Lypd1/Morc1/Npas2/Pde8b	9	1
BP	GO:0032922	circadian regulation of gene expression	10/736	0.007461	Arntl/Ciart/Cry2/Mycbp2/Npas2/Per1/Per2/Per3/Rorc/Usp2	10	1.264911
BP	GO:0043153	entrainment of circadian clock by photoperiod	6/736	0.008429	Cry2/Fbxl21/Per1/Per2/Per3/Usp2	6	1.632993
BP	GO:0035821	modification of morphology or physiology of other organism	16/736	0.008771	Anxa2/Cxcl5/Defa17/Defa2/Defa21/Defa22/Defa23/Defa24/Defa3/Defa30/Defa31/Defa34/Defa5/Ifng/Ltf/Nos2	16	3
BP	GO:0042596	fear response	9/736	0.009989	Eif4g1/Fbxl20/Gja1/Gng7/Kalrn/Lypd1/Morc1/Npas2/Pde8b	9	1
BP	GO:0009648	photoperiodism	6/736	0.009989	Cry2/Fbxl21/Per1/Per2/Per3/Usp2	6	1.632993
BP	GO:0035461	vitamin transmembrane transport	5/736	0.011123	Slc19a1/Slc19a2/Slc19a3/Slc5a6/Stra6l	5	2.236068
BP	GO:0010959	regulation of metal ion transport	29/736	0.012466	Amigo1/Ank3/Anxa2/Asph/Cckar/Ccl12/Coro1a/Dpp6/G6pdx/Gck/Gja1/Gnas/Gpr35/Ifng/Igf1/Kcne3/Kcnq1/Neto1/Neto2/Nkain4/P2rx7/Pde4d/Per1/Pla2g6/Pln/Sgk1/Tmbim6/Tmc1/Usp2	29	-0.185695
BP	GO:0051056	regulation of small GTPase mediated signal transduction	21/736	0.012466	Abr/Adra1b/Arfgef1/Arhgap24/Arhgef3/Arhgef39/Arpp19/Cyth2/Git1/Gpr35/Igf1/Iqsec2/Itsn2/Kalrn/Mfn2/Ntrk1/Plekhg2/Plekhg5/Rasgef1a/Spata13/Syngap1	21	-0.654654
BP	GO:0009649	entrainment of circadian clock	6/736	0.014649	Cry2/Fbxl21/Per1/Per2/Per3/Usp2	6	1.632993
BP	GO:0032651	regulation of interleukin-1 beta production	10/736	0.015032	Casp8/Hk1/Ifi204/Ifi211/Ifng/Igf1/Nlrp3/P2rx7/Sirpa/Tlr8	10	1.264911
BP	GO:0007623	circadian rhythm	18/736	0.015449	Arntl/Ciart/Cipc/Cry2/Dbp/Fbxl21/Igf1/Mycbp2/Nos2/Npas2/Nr1d2/Ntrk1/Per1/Per2/Per3/Rorc/Timeless/Usp2	18	0.942809
BP	GO:0032731	positive regulation of	8/736	0.015449	Casp8/Hk1/Ifi204/Ifi211/Ifng/Nlrp3/P2rx7/Tlr8	8	1.414214

		interleukin-1 beta production					
BP	GO:0015914	phospholipid transport	9/736	0.019392	Abcb1a/Abcb4/Atp11a/Atp8b4/Atp9b/P2rx7/Pltp/Prelid3b/Prkcd	9	0.333333
BP	GO:0009410	response to xenobiotic stimulus	19/736	0.019392	Abcb4/Adgrl3/Adra1b/Akr1c12/Akr1c13/Arnt2/Cyp2j8/Cyp2s1/Cyp46a1/Dlg4/E2f1/Kalrn/Pck1/Pgr/Rorc/Ryr1/Stat5a/Sult1a1/Ugt1a6a	19	2.064742
BP	GO:0045332	phospholipid translocation	6/736	0.019392	Abcb1a/Abcb4/Atp11a/Atp8b4/Atp9b/P2rx7	6	0
BP	GO:0040013	negative regulation of locomotion	24/736	0.019392	Abhd6/Abr/Adora3/Adtrp/Angpt4/Ccl12/Cd300a/Dcn/Dusp10/Dusp22/Dusp3/Epha1/Il1rn/Il33/Krit1/Mctp1/Meox2/Myocd/Pbld1/Sema3f/Sema4a/Sema4c/Sema6c/Stard13	24	1.632993
BP	GO:0046889	positive regulation of lipid biosynthetic process	11/736	0.01956	Abhd6/Adgrf5/Cyp17a1/Htr2b/Ifng/Igf1/Nr1h2/Pla2g6/Por/Prkcd/Slc27a1	11	0.904534
BP	GO:0002294	CD4-positive, alpha-beta T cell differentiation involved in immune response	9/736	0.019654	Foxp1/Ifng/Il18r1/Il23a/Il27/Myb/Nlrp3/Rorc/Sema4a	9	2.333333
BP	GO:0002293	alpha-beta T cell differentiation involved in immune response	9/736	0.02124	Foxp1/Ifng/Il18r1/Il23a/Il27/Myb/Nlrp3/Rorc/Sema4a	9	2.333333
BP	GO:0048640	negative regulation of developmental growth	13/736	0.02124	Cdkn1b/Dusp10/Fgfr3/Foxp1/G6pdx/Gja1/Gnas/Mag/Sema3f/Sema4a/Sema4c/Sema6c/Spg20	13	0.27735
BP	GO:0034204	lipid translocation	6/736	0.02124	Abcb1a/Abcb4/Atp11a/Atp8b4/Atp9b/P2rx7	6	0
BP	GO:0097035	regulation of membrane lipid distribution	8/736	0.021697	Abcb1a/Abcb4/Atp11a/Atp8b4/Atp9b/P2rx7/Plscr2/Prkcd	8	0.707107
BP	GO:0002287	alpha-beta T cell activation involved in immune response	9/736	0.022369	Foxp1/Ifng/Il18r1/Il23a/Il27/Myb/Nlrp3/Rorc/Sema4a	9	2.333333
BP	GO:0007409	axonogenesis	30/736	0.024181	Ablim1/Amigo1/Ank3/Apbb2/Artn/Bsg/Cckar/Chodl/Dab1/Eif4g2/Epha1/Fgfr3/Flrt2/Foxp1/Golga4/Kalrn/Mag/Mapt/Mycbp2/Ntrk1/Numb/Sema3f/Sema4a/Sema4c/Sema6c/Spg20/Syngap1/Trpc5/Tsku/Zswim8	30	-0.365148
BP	GO:0015748	organophosphate ester transport	10/736	0.02497	Abcb1a/Abcb4/Atp11a/Atp8b4/Atp9b/Gja1/P2rx7/Pltp/Prelid3b/Prkcd	10	0.632456

BP	GO:0044419	interspecies interaction between organisms	27/736	0.026508	Anxa2/Cul1/Cxcl5/Defa17/Defa2/Defa21/Defa22/Defa23/Defa24/Defa3/Defa30/Defa31/Defa34/Defa5/Hspd1/Ifitm3/Ifng/Irgm1/Ltf/Nlrx1/Nos2/Ppih/Slc20a2/Snx3/Trim10/Ube2i/Vcam1	27	2.501851
BP	GO:0045821	positive regulation of glycolytic process	5/736	0.030007	Entpd5/Gck/Ifng/Igf1/P2rx7	5	0.447214
BP	GO:0032732	positive regulation of interleukin-1 production	8/736	0.031913	Casp8/Hk1/Ifi204/Ifi211/Ifng/Nlrp3/P2rx7/Tlr8	8	1.414214
BP	GO:0000082	G1/S transition of mitotic cell cycle	15/736	0.031913	Bid/Ccl12/Ccnd2/Ccne1/Cdkn1b/Ctdspl/E2f1/E2f7/Eif4g1/Fbxo7/Gigyf2/Myb/Phf8/Tcf19/Ube2e2	15	2.32379
BP	GO:0030336	negative regulation of cell migration	20/736	0.031913	Abhd6/Abr/Adora3/Adtrp/Angpt4/Ccl12/Cd300a/Dcn/Dusp10/Dusp22/Dusp3/Epha1/Il1rn/Il33/Krit1/Mctp1/Meox2/Myocd/Pbld1/Stard13	20	1.341641
BP	GO:0032611	interleukin-1 beta production	10/736	0.031913	Casp8/Hk1/Ifi204/Ifi211/Ifng/Igf1/Nlrp3/P2rx7/Sirpa/Tlr8	10	1.264911
BP	GO:0002292	T cell differentiation involved in immune response	9/736	0.031913	Foxp1/Ifng/Il18r1/Il23a/Il27/Myb/Nlrp3/Rorc/Sema4a	9	2.333333
BP	GO:0009166	nucleotide catabolic process	13/736	0.031913	Art2b/Dera/Enpp5/Entpd5/Gck/Hk1/Ifng/Igf1/Nudt1/P2rx7/Pde4d/Pfkfb2/Prkag2	13	-0.27735
BP	GO:0045666	positive regulation of neuron differentiation	29/736	0.031913	Amigo1/Chodl/Cpeb3/Cxcl5/Dab1/Dlg4/Eif4g1/Eif4g2/Elavl4/Golga4/Heyl/Hmg20b/Ifng/Irx3/Itsn2/Kalrn/Mapk6/Mapt/Mob2/Ntrk1/Numb/Parp6/Rnf157/Sgk1/Snx3/Styxl1/Tgif1/Trpc5/Ube2v2	29	0.185695
BP	GO:0046434	organophosphate catabolic process	17/736	0.031913	Abhd6/Art2b/Dera/Enpp5/Entpd5/Gck/Hk1/Ifng/Igf1/Inpp5e/Mtmr2/Nudt1/P2rx7/Pde4d/Pfkfb2/Prkag2/Prkcd	17	0.727607
BP	GO:0030813	positive regulation of nucleotide catabolic process	5/736	0.032174	Entpd5/Gck/Ifng/Igf1/P2rx7	5	0.447214
BP	GO:0051197	positive regulation of coenzyme metabolic process	5/736	0.032174	Entpd5/Gck/Ifng/Igf1/P2rx7	5	0.447214
BP	GO:0032102	negative regulation of response to external stimulus	23/736	0.037025	Abr/Adtrp/Anxa2/Ccl12/Cdkn1b/Chid1/Dusp10/Dusp3/Ets1/Gja1/Igf1/Ltf/Nlrp3/Nlrx1/Nr1d2/Nucb2/Prkcd/	23	1.459601

					Sema3f/Sema4a/Sema4c/Sema6c/Sirpa/Tfpi		
BP	GO:0042752	regulation of circadian rhythm	12/736	0.037557	Arntl/Cipc/Cry2/Fbxl21/Npas2/Nr1d2/Per1/Per2/Per3/Rorc/Timeless/Usp2	12	1.732051
BP	GO:0034248	regulation of cellular amide metabolic process	25/736	0.040472	Cnot2/Cnot6l/Cnot8/Cpeb3/Eif4ebp3/Eif4g1/Eif4g2/Elavl4/Gigyf2/Ifng/Igf1/Inpp5e/Kbtbd8/Larp4b/Mif4gd/Ormdl2/Pdk4/Per1/Per2/Pla2g6/Prkcd/Pstk/Rbm3/Samd4/Spon1	25	2.2
BP	GO:0009142	nucleoside triphosphate biosynthetic process	14/736	0.042653	Adk/Atp5c1/Atp5h/Cad/Ctps2/Entpd5/Gck/Hk1/Ifng/Igf1/Nme4/P2rx7/Pfkfb2/Prkag2	14	0.534522
BP	GO:0032652	regulation of interleukin-1 production	10/736	0.043391	Casp8/Hk1/Ifi204/Ifi211/Ifng/Igf1/Nlrp3/P2rx7/Sirpa/Tlr8	10	1.264911
BP	GO:0046390	ribose phosphate biosynthetic process	18/736	0.045641	Ampd2/Ampd3/Aprt/Atp5c1/Atp5h/Cad/Ctps2/Entpd5/G6pdx/Gck/Hk1/Ifng/Igf1/Nme4/P2rx7/Pdk4/Pfkfb2/Prkag2	18	0.942809
BP	GO:0008202	steroid metabolic process	21/736	0.045715	Acadvl/Akr1c12/Akr1c13/Akr1c14/Cel/Cyp17a1/Cyp3a25/Cyp46a1/Dhcr7/G6pdx/Hsd17b2/Ifng/Igf1/Pde8b/Por/Rorc/Sgpl1/Stat5a/Sult1a1/Sult2b1/Thrb	21	1.963961
BP	GO:2000146	negative regulation of cell motility	20/736	0.046784	Abhd6/Abr/Adora3/Adtrp/Angpt4/Ccl12/Cd300a/Dcn/Dusp10/Dusp22/Dusp3/Epha1/Il1rn/Il33/Krit1/Mctp1/Meox2/Myocd/Pbld1/Stard13	20	1.341641
BP	GO:0044262	cellular carbohydrate metabolic process	20/736	0.051949	Adra1b/Arpp19/B3gnt8/Extl2/Gaa/Gck/Gnptg/Hk1/Igf1/Inpp5e/Itpk1/Mtmr2/Pck1/Pdk4/Per2/Pfkfb2/Plcd1/Pomc/Prkag2/Rorc	20	1.341641
BP	GO:1903039	positive regulation of leukocyte cell-cell adhesion	16/736	0.051949	Adk/Coro1a/Dusp10/Ets1/Hspd1/Ifng/Igf1/Il23a/Myb/Nlrp3/Sirpa/Spta1/Stat5a/Vcam1/Zap70/Zbtb16	16	1
BP	GO:0032878	regulation of establishment or maintenance of cell polarity	5/736	0.052027	Arfgef1/Bcas3/Igf1/Krit1/Wdpcp	5	0.447214
BP	GO:0042093	T-helper cell differentiation	8/736	0.052027	Foxp1/Il18r1/Il23a/Il27/Myb/Nlrp3/Rorc/Sema4a	8	2.12132
BP	GO:0009165	nucleotide biosynthetic process	20/736	0.052027	Adk/Ampd2/Ampd3/Aprt/Atp5c1/Atp5h/Cad/Ctps2/Entpd5/G6pdx/Gck/Hk1/Ifng/Igf1/Nme4/Nos2/P2rx7/Pdk4/Pfkfb2/Prkag2	20	0.447214
BP	GO:0044843	cell cycle G1/S phase	15/736	0.052547	Bid/Ccl12/Ccnd2/Ccne1/Cdkn1b/Ctdspl/E2f1/E2f7/Eif4	15	2.32379

		transition			g1/Fbxo7/Gigyf2/Myb/Phf8/Tcf19/Ube2e2		
BP	GO:0046632	alpha-beta T cell differentiation	11/736	0.052547	Foxp1/Ifng/Il18r1/Il23a/Il27/Myb/Nlrp3/Rorc/Sema4a/Zap70/Zbtb16	11	2.110579
BP	GO:0050770	regulation of axonogenesis	16/736	0.052547	Amigo1/Chodl/Dab1/Eif4g2/Golga4/Mag/Mapt/Mycbp2/Sema3f/Sema4a/Sema4c/Sema6c/Spg20/Syngap1/Trpc5/Zswim8	16	0
BP	GO:1901292	nucleoside phosphate catabolic process	13/736	0.052547	Art2b/Dera/Enpp5/Entpd5/Gck/Hk1/Ifng/Igf1/Nudt1/P2rx7/Pde4d/Pfkfb2/Prkag2	13	-0.27735
BP	GO:1903037	regulation of leukocyte cell-cell adhesion	20/736	0.052547	Adk/Adtrp/Cd300a/Coro1a/Dusp10/Dusp22/Dusp3/Ets1/Hspd1/Ifng/Igf1/Il23a/Myb/Nlrp3/Sirpa/Spta1/Stat5a/Vcam1/Zap70/Zbtb16	20	0.447214
BP	GO:0009914	hormone transport	24/736	0.052547	Abcb1a/Adora3/Arntl/Bmp8a/Cckar/Cry2/Gck/Gip/Gja1/Gnas/Ifng/Il1rn/Kalrn/Kcnq1/Ly6e/Nnat/Nos2/Nucb2/Pde8b/Per2/Pfkfb2/Pla2g6/Pomc/Ttr	24	1.224745
BP	GO:0050870	positive regulation of T cell activation	15/736	0.054166	Adk/Coro1a/Dusp10/Hspd1/Ifng/Igf1/Il23a/Myb/Nlrp3/Sirpa/Spta1/Stat5a/Vcam1/Zap70/Zbtb16	15	0.774597
BP	GO:0002697	regulation of immune effector process	26/736	0.054166	Abr/Adora3/Cd300a/Cd55/Cxcl5/Dusp10/Dusp22/Fcgr1/Foxp1/H2-T22/H2-T3/Hk1/Hspd1/Ifng/Il18r1/Il23a/Il27/Il33/Myb/Myo18a/Nlrp3/Nlrx1/P2rx7/Spon2/Stat5a/Tmbim6	26	1.176697
BP	GO:0030810	positive regulation of nucleotide biosynthetic process	6/736	0.054166	Entpd5/Gck/Ifng/Igf1/Nos2/P2rx7	6	0
BP	GO:0033144	negative regulation of intracellular steroid hormone receptor signaling pathway	6/736	0.054166	Arntl/Cnot2/Cry2/Heyl/Igf1/Per1	6	-0.816497
BP	GO:1900373	positive regulation of purine nucleotide biosynthetic process	6/736	0.054166	Entpd5/Gck/Ifng/Igf1/Nos2/P2rx7	6	0
BP	GO:0009201	ribonucleoside triphosphate biosynthetic process	13/736	0.054166	Atp5c1/Atp5h/Cad/Ctps2/Entpd5/Gck/Hk1/Ifng/Igf1/Nme4/P2rx7/Pfkfb2/Prkag2	13	0.83205
BP	GO:2000045	regulation of G1/S transition of mitotic cell cycle	11/736	0.054166	Bid/Ccl12/Ccnd2/Ccne1/Ctdspl/E2f1/E2f7/Eif4g1/Fbxo7/Gigyf2/Ube2e2	11	1.507557

BP	GO:0031331	positive regulation of cellular catabolic process	23/736	0.055208	Adra1b/Atxn3/Aurka/Bid/Cnot6l/Cnot8/Cpeb3/Dcn/Entpd5/Fbxo7/Gck/Gigyf2/Git1/Herpud1/Ifng/Igf1/Il33/Irgm1/Mfn2/P2rx7/Prkcd/Ube2v2/Wac	23	0.625543
BP	GO:0043367	CD4-positive, alpha-beta T cell differentiation	9/736	0.055208	Foxp1/Ifng/Il18r1/Il23a/Il27/Myb/Nlrp3/Rorc/Sema4a	9	2.333333
BP	GO:1901293	nucleoside phosphate biosynthetic process	20/736	0.055338	Adk/Ampd2/Ampd3/Aprt/Atp5c1/Atp5h/Cad/Ctps2/Entpd5/G6pdx/Gck/Hk1/Ifng/Igf1/Nme4/Nos2/P2rx7/Pdk4/Pfkfb2/Prkag2	20	0.447214
BP	GO:0033555	multicellular organismal response to stress	10/736	0.061115	Eif4g1/Fbxl20/Gja1/Gng7/Kalrn/Lypd1/Morc1/Npas2/Ntrk1/Pde8b	10	0.632456
BP	GO:0050771	negative regulation of axonogenesis	8/736	0.061584	Dab1/Mag/Sema3f/Sema4a/Sema4c/Sema6c/Spg20/Syngap1	8	0
BP	GO:0009260	ribonucleotide biosynthetic process	17/736	0.061584	Ampd2/Ampd3/Aprt/Atp5c1/Atp5h/Cad/Ctps2/Entpd5/Gck/Hk1/Ifng/Igf1/Nme4/P2rx7/Pdk4/Pfkfb2/Prkag2	17	1.212678
BP	GO:1903531	negative regulation of secretion by cell	17/736	0.061584	Abr/Adtrp/Bmp8a/Cd300a/Cry2/Erp29/Gck/Il1rn/Il33/Kalrn/Kcnq1/Nlrp3/Nucb2/Pde8b/Tcp11/Tlr8/Tmbim6	17	1.212678
BP	GO:0035710	CD4-positive, alpha-beta T cell activation	10/736	0.063267	Cdh26/Foxp1/Ifng/Il18r1/Il23a/Il27/Myb/Nlrp3/Rorc/Sema4a	10	1.897367
BP	GO:0007626	locomotory behavior	18/736	0.063665	Adgrl3/Adra1b/Ccnd2/Ciart/Dab1/Dlg4/Dmrt3/Elavl4/Espn/Gaa/Gigyf2/Gip/Gng7/Kalrn/Lgi4/Mapt/Npas2/Usp2	18	0
BP	GO:0097009	energy homeostasis	6/736	0.063665	Ampd2/Ampd3/Bmp8a/Eif4g1/Irx3/Nr1d2	6	0
BP	GO:0010256	endomembrane system organization	24/736	0.063665	Acrbp/Ank3/Arfgef1/Atp8b4/Bcas3/Camsap3/Col5a1/Dctn1/Dnajc13/Myo18a/Nsfl1c/Nup93/P2rx7/Parp11/Plscr2/Prkcd/Snx3/Spta1/Stx16/Stx18/Tor1aip2/Trappc12/Ubl4a/Vmp1	24	0.408248
BP	GO:0046835	carbohydrate phosphorylation	4/736	0.063665	Gck/Gnptg/Hk1/Itpk1	4	1
BP	GO:0045913	positive regulation of carbohydrate metabolic process	9/736	0.063835	Adra1b/Arpp19/Entpd5/Gck/Ifng/Igf1/P2rx7/Pfkfb2/Plcd1	9	0.333333
BP	GO:0009163	nucleoside biosynthetic process	5/736	0.065651	Adk/Aprt/Cad/Ctps2/Nme4	5	1.341641

BP	GO:0042455	ribonucleoside biosynthetic process	5/736	0.065651	Adk/Aprt/Cad/Ctps2/Nme4	5	1.341641
BP	GO:0019218	regulation of steroid metabolic process	10/736	0.06734	Acadvl/Cyp17a1/Dhcr7/Ifng/Igf1/Pde8b/Por/Rorc/Stat5a/Thrb	10	1.264911
BP	GO:0032370	positive regulation of lipid transport	8/736	0.06734	Abcb4/Anxa2/Nr1h2/Nucb2/P2rx7/Pla2g6/Pltp/Prkcd	8	0
BP	GO:0045834	positive regulation of lipid metabolic process	13/736	0.067526	Abhd6/Adgrf5/Cyp17a1/Fgfr3/Htr2b/Ifng/Igf1/Nr1h2/Nucb2/Pla2g6/Por/Prkcd/Slc27a1	13	0.83205
BP	GO:0046631	alpha-beta T cell activation	13/736	0.067526	Cd300a/Cdh26/Foxp1/Ifng/Il18r1/Il23a/Il27/Myb/Nlrp3/Rorc/Sema4a/Zap70/Zbtb16	13	1.941451
BP	GO:0008654	phospholipid biosynthetic process	13/736	0.06955	Adgrf5/Agpat1/Agpat4/Chkb/Far1/Htr2b/Idi1/Lclat1/Pgap2/Pigp/Pik3cd/Pla2g6/Slc27a1	13	1.38675
BP	GO:0009127	purine nucleoside monophosphate biosynthetic process	13/736	0.06955	Ampd2/Ampd3/Aprt/Atp5c1/Atp5h/Entpd5/Gck/Hk1/Ifng/Igf1/P2rx7/Pfkfb2/Prkag2	13	0.27735
BP	GO:0009168	purine ribonucleoside monophosphate biosynthetic process	13/736	0.06955	Ampd2/Ampd3/Aprt/Atp5c1/Atp5h/Entpd5/Gck/Hk1/Ifng/Igf1/P2rx7/Pfkfb2/Prkag2	13	0.27735
BP	GO:0002699	positive regulation of immune effector process	18/736	0.069773	Adora3/Cd300a/Fcgr1/Foxp1/H2-T22/H2-T3/Hk1/Hspd1/Ifng/Il18r1/Il23a/Il33/Myb/Myo18a/Nlrp3/P2rx7/Spon2/Stat5a	18	1.414214
BP	GO:0042445	hormone metabolic process	16/736	0.070276	Akr1c14/Awat2/Cyp17a1/Cyp2s1/Cyp46a1/Dhcr7/Dio1/Hsd17b2/Ifng/Igf1/Pde8b/Por/Sgpl1/Slco4a1/Sult1a1/Ttr	16	2
BP	GO:0043470	regulation of carbohydrate catabolic process	7/736	0.070276	Adra1b/Entpd5/Gck/Ifng/Igf1/P2rx7/Prkag2	7	0.377964
BP	GO:0051194	positive regulation of cofactor metabolic process	5/736	0.070276	Entpd5/Gck/Ifng/Igf1/P2rx7	5	0.447214
BP	GO:0048588	developmental cell growth	18/736	0.070276	Adra1b/Aurka/Eif4g2/Foxp1/G6pdx/Golga4/Igf1/Itsn2/Mag/Mapt/Myocd/Rnf157/Sema3f/Sema4a/Sema4c/Sema6c/Spg20/Trpc5	18	1.885618
BP	GO:0032612	interleukin-1 production	10/736	0.070537	Casp8/Hk1/Ifi204/Ifi211/Ifng/Igf1/Nlrp3/P2rx7/Sirpa/Tlr8	10	1.264911

BP	GO:0006164	purine nucleotide biosynthetic process	17/736	0.071348	Adk/Ampd2/Ampd3/Aprt/Atp5c1/Atp5h/Entpd5/Gck/Hk1/Ifng/Igf1/Nme4/Nos2/P2rx7/Pdk4/Pfkfb2/Prkag2	17	0.242536
BP	GO:0051048	negative regulation of secretion	18/736	0.071348	Abr/Adtrp/Bmp8a/Cd300a/Cry2/Erp29/Gck/Gnas/Il1rn/Il33/Kalrn/Kcnq1/Nlrp3/Nucb2/Pde8b/Tcp11/Tlr8/Tmbim6	18	0.942809
BP	GO:0050863	regulation of T cell activation	20/736	0.071348	Adk/Cd300a/Coro1a/Dusp10/Dusp22/Dusp3/Hspd1/Ifng/Igf1/Il23a/Il27/Myb/Nlrp3/Rorc/Sirpa/Spta1/Stat5a/Vcam1/Zap70/Zbtb16	20	0.894427
BP	GO:0051196	regulation of coenzyme metabolic process	7/736	0.073959	Entpd5/Gck/Ifng/Igf1/P2rx7/Pdk4/Prkag2	7	0.377964
BP	GO:0006112	energy reserve metabolic process	9/736	0.073959	Adgrf5/Adra1b/Gaa/Gck/Gnas/Igf1/Per2/Pomc/Prkag2	9	-0.333333
BP	GO:0022409	positive regulation of cell-cell adhesion	17/736	0.075354	Adk/Ank3/Coro1a/Dusp10/Ets1/Hspd1/Ifng/Igf1/Il23a/Myb/Nlrp3/Sirpa/Spta1/Stat5a/Vcam1/Zap70/Zbtb16	17	0.727607
BP	GO:1902235	regulation of endoplasmic reticulum stress-induced intrinsic apoptotic signaling pathway	5/736	0.076752	Bok/Erp29/Herpud1/Rnf183/Tmbim6	5	-1.341641
BP	GO:0009156	ribonucleoside monophosphate biosynthetic process	13/736	0.077131	Ampd2/Ampd3/Aprt/Atp5c1/Atp5h/Entpd5/Gck/Hk1/Ifng/Igf1/P2rx7/Pfkfb2/Prkag2	13	0.27735
BP	GO:0031346	positive regulation of cell projection organization	27/736	0.078467	Amigo1/Arap1/Bcas3/Cep135/Chodl/Cpeb3/Cxcl5/Dlg4/Eif4g2/Elavl4/Espn/Golga4/Itsn2/Kalrn/Mapk6/Mapt/Mob2/Ntrk1/Numb/P2rx7/Parp6/Rnf157/Sgk1/Snx3/Styxl1/Trpc5/Ube2v2	27	0.19245
BP	GO:0006869	lipid transport	21/736	0.078594	Abcb1a/Abcb4/Anxa2/Atp11a/Atp8b4/Atp9b/Cry2/Gulp1/Nme4/Nos2/Nr1h2/Nucb2/P2rx7/Pla2g6/Plin2/Pltp/Pomc/Prelid3b/Prkcd/Slc27a1/Stra6l	21	1.963961
BP	GO:0043094	cellular metabolic compound salvage	4/736	0.08014	Adk/Ampd2/Ampd3/Aprt	4	0
BP	GO:0072522	purine-containing compound biosynthetic process	17/736	0.081497	Adk/Ampd2/Ampd3/Aprt/Atp5c1/Atp5h/Entpd5/Gck/Hk1/Ifng/Igf1/Nme4/Nos2/P2rx7/Pdk4/Pfkfb2/Prkag2	17	0.242536
BP	GO:0009896	positive regulation of	25/736	0.081497	Adra1b/Atxn3/Aurka/Bid/Cnot6l/Cnot8/Cpeb3/Dcn/En	25	0.6

		catabolic process			tpd5/Fbxo7/Gck/Gigyf2/Git1/Gja1/Gpc3/Herpud1/Ifng/Igf1/Il33/Irgm1/Mfn2/P2rx7/Prkcd/Ube2v2/Wac		
BP	GO:0006643	membrane lipid metabolic process	13/736	0.081998	Cel/Cerk/Fam57b/Hacd4/Map7/Ormdl2/P2rx7/Pgap2/Pigp/Pla2g6/Prkcd/Sgpl1/St3gal4	13	1.941451
BP	GO:0010043	response to zinc ion	5/736	0.083477	Mt1/Mt2/P2rx7/Pln/Slc30a2	5	1.341641
BP	GO:0002532	production of molecular mediator involved in inflammatory response	8/736	0.083477	Adora3/Cd300a/Chid1/Dusp10/Il17c/Nos2/Per1/Sirpa	8	0.707107
BP	GO:0048638	regulation of developmental growth	24/736	0.083477	Cdkn1b/Dusp10/Eif4g2/Fgf9/Fgfr3/Foxp1/G6pdx/Gja1/Gnas/Golga4/Igf1/Itsn2/Mag/Mapt/Por/Rnf157/Sema3f/Sema4a/Sema4c/Sema6c/Sgpl1/Spg20/Stat5a/Trpc5	24	1.224745
BP	GO:0031345	negative regulation of cell projection organization	15/736	0.083477	Arhgap24/Cep97/Cyth2/Dab1/Fat3/Mag/Prkcd/Sema3f/Sema4a/Sema4c/Sema6c/Spg20/Syngap1/Tbx6/Trpc5	15	-0.258199
BP	GO:0044772	mitotic cell cycle phase transition	22/736	0.083477	Abcb1a/Arpp19/Bid/Ccl12/Ccnd2/Ccne1/Cdkn1b/Chek1/Ctdspl/Donson/E2f1/E2f7/Eif4g1/Fbxl12/Fbxo7/Gigyf2/Kat14/Myb/Npm2/Phf8/Tcf19/Ube2e2	22	2.558409
BP	GO:0034655	nucleobase-containing compound catabolic process	25/736	0.083477	Apobec1/Art2b/Axin2/Cnot6l/Cnot8/Cpeb3/Dera/E2f1/Elavl4/Enpp5/Entpd5/Gck/Gigyf2/Hk1/Ifng/Igf1/Nudt1/Oas2/P2rx7/Pde4d/Pfkfb2/Pkp1/Prkag2/Rbm8a/Samd4	25	1
BP	GO:0045806	negative regulation of endocytosis	7/736	0.083477	Cd300a/Dlg4/Mctp1/Mtmr2/Nr1h2/Sirpa/Snx3	7	1.133893
BP	GO:0030808	regulation of nucleotide biosynthetic process	8/736	0.083477	Entpd5/Gck/Ifng/Igf1/Nos2/P2rx7/Pdk4/Prkag2	8	0
BP	GO:0042446	hormone biosynthetic process	8/736	0.083477	Cyp17a1/Dhcr7/Dio1/Hsd17b2/Ifng/Igf1/Pde8b/Por	8	0
BP	GO:1900371	regulation of purine nucleotide biosynthetic process	8/736	0.083477	Entpd5/Gck/Ifng/Igf1/Nos2/P2rx7/Pdk4/Prkag2	8	0
BP	GO:1902806	regulation of cell cycle G1/S phase transition	11/736	0.083477	Bid/Ccl12/Ccnd2/Ccne1/Ctdspl/E2f1/E2f7/Eif4g1/Fbxo7/Gigyf2/Ube2e2	11	1.507557
BP	GO:0019693	ribose phosphate metabolic process	26/736	0.083477	Acsm5/Ampd2/Ampd3/Aprt/Atp5c1/Atp5h/Bid/Cad/Ctps2/Entpd5/Far1/G6pdx/Gck/Glyat/H6pd/Hk1/Ifng/Ig	26	1.568929

					f1/Ndufv2/Nme4/P2rx7/Pde4d/Pdk4/Pfkfb2/Prkag2/Slt2b1		
BP	GO:1901657	glycosyl compound metabolic process	10/736	0.083477	Adk/Akr1b8/Akr1c14/Ampd2/Ampd3/Aprt/Cad/Ctps2/Dera/Nme4	10	1.264911
BP	GO:0090276	regulation of peptide hormone secretion	16/736	0.083477	Arntl/Bmp8a/Cckar/Gck/Gip/Gja1/Ifng/Kalrn/Kcnq1/Nnat/Nos2/Nucb2/Pde8b/Per2/Pfkfb2/Pla2g6	16	0.5
BP	GO:0062012	regulation of small molecule metabolic process	22/736	0.083477	Acadvl/Adra1b/Arpp19/Dhcr7/Entpd5/Gck/Gip/Ifng/Igf1/Nos2/Nr1h2/Nucb2/P2rx7/Pdk4/Pfkfb2/Plcd1/Pomc/Por/Prkag2/Rorc/Sirt5/Thrb	22	1.279204
BP	GO:0032011	ARF protein signal transduction	4/736	0.083477	Arfgef1/Cyth2/Git1/Iqsec2	4	-1
BP	GO:0032012	regulation of ARF protein signal transduction	4/736	0.083477	Arfgef1/Cyth2/Git1/Iqsec2	4	-1
BP	GO:0046886	positive regulation of hormone biosynthetic process	4/736	0.083477	Cyp17a1/Ifng/Igf1/Por	4	0
BP	GO:0090030	regulation of steroid hormone biosynthetic process	4/736	0.083477	Cyp17a1/Igf1/Pde8b/Por	4	0
BP	GO:2000114	regulation of establishment of cell polarity	4/736	0.083477	Arfgef1/Bcas3/Krit1/Wdpcp	4	1
BP	GO:0042742	defense response to bacterium	25/736	0.083477	Cxcl5/Defa17/Defa2/Defa21/Defa22/Defa23/Defa24/Defa3/Defa30/Defa31/Defa34/Defa5/Fcgr1/Gbp10/Ifng/Iigp1/Il23a/Ltf/Lyz1/Nlrp3/Nos2/P2rx7/Pla2g6/Prkcd/Spon2	25	3.8
BP	GO:0050718	positive regulation of interleukin-1 beta secretion	5/736	0.083477	Hk1/Ifng/Nlrp3/P2rx7/Tlr8	5	1.341641
BP	GO:1901659	glycosyl compound biosynthetic process	5/736	0.083477	Adk/Aprt/Cad/Ctps2/Nme4	5	1.341641
BP	GO:0009145	purine nucleoside triphosphate biosynthetic process	12/736	0.083477	Adk/Atp5c1/Atp5h/Entpd5/Gck/Hk1/Ifng/Igf1/Nme4/P2rx7/Pfkfb2/Prkag2	12	0
BP	GO:0009124	nucleoside monophosphate	13/736	0.084008	Ampd2/Ampd3/Aprt/Atp5c1/Atp5h/Entpd5/Gck/Hk1/I	13	0.27735

		biosynthetic process			fng/Igf1/P2rx7/Pfkfb2/Prkag2		
BP	GO:2001257	regulation of cation channel activity	13/736	0.084008	Amigo1/Ank3/Asph/Dlg4/Gpr35/Ifng/Kcne3/Kcnq1/Neto1/Neto2/Pde4d/Pla2g6/Pln	13	0.27735
BP	GO:0032409	regulation of transporter activity	18/736	0.084008	Abcb1a/Amigo1/Ank3/Asph/Dlg4/Gja1/Gopc/Gpr35/Hk1/Ifng/Kcne3/Kcnq1/Neto1/Neto2/Pde4d/Pla2g6/Pln/Prkcd	18	0.942809
BP	GO:0003254	regulation of membrane depolarization	6/736	0.0847	Ank3/Bok/Dcn/Gja1/Hsh2d/P2rx7	6	0
BP	GO:0010976	positive regulation of neuron projection development	22/736	0.087006	Amigo1/Chodl/Cpeb3/Cxcl5/Dlg4/Eif4g2/Elavl4/Golga4/Itsn2/Kalrn/Mapk6/Mapt/Mob2/Ntrk1/Numb/Parp6/Rnf157/Sgk1/Snx3/Styxl1/Trpc5/Ube2v2	22	0.426401
BP	GO:0007589	body fluid secretion	10/736	0.088554	Adora3/Anxa2/Aprt/Cckar/Cel/Gja1/Kalrn/Nr1h2/Oas2/Stat5a	10	1.264911
BP	GO:0008360	regulation of cell shape	12/736	0.089172	Anxa7/Arap1/Arhgap15/Ccl12/Ccl24/Coro1a/Epb41l2/Fmnl2/Sema4a/Sprr2a1/Spta1/Syne3	12	-0.57735
BP	GO:0007159	leukocyte cell-cell adhesion	20/736	0.089322	Adk/Adtrp/Cd300a/Coro1a/Dusp10/Dusp22/Dusp3/Ets1/Hspd1/Ifng/Igf1/Il23a/Myb/Nlrp3/Sirpa/Spta1/Stat5a/Vcam1/Zap70/Zbtb16	20	0.447214
BP	GO:0051180	vitamin transport	5/736	0.089322	Slc19a1/Slc19a2/Slc19a3/Slc5a6/Stra6l	5	2.236068
BP	GO:2001171	positive regulation of ATP biosynthetic process	5/736	0.089322	Entpd5/Gck/Ifng/Igf1/P2rx7	5	0.447214
BP	GO:0006110	regulation of glycolytic process	6/736	0.089322	Entpd5/Gck/Ifng/Igf1/P2rx7/Prkag2	6	0
BP	GO:0030811	regulation of nucleotide catabolic process	6/736	0.089322	Entpd5/Gck/Ifng/Igf1/P2rx7/Prkag2	6	0
BP	GO:1902667	regulation of axon guidance	6/736	0.089322	Mycbp2/Sema3f/Sema4a/Sema4c/Sema6c/Zswim8	6	0
BP	GO:0009209	pyrimidine ribonucleoside triphosphate biosynthetic process	3/736	0.089322	Cad/Ctps2/Nme4	3	1.732051
BP	GO:2000303	regulation of ceramide biosynthetic process	3/736	0.089322	Ormdl2/Pla2g6/Prkcd	3	0.57735
BP	GO:2000644	regulation of receptor catabolic process	3/736	0.089322	Anxa2/Git1/Mtmr2	3	-0.57735

BP	GO:0046879	hormone secretion	22/736	0.089322	Adora3/Arntl/Bmp8a/Cckar/Cry2/Gck/Gip/Gja1/Gnas/Ifng/Il1rn/Kalrn/Kcnq1/Ly6e/Nnat/Nos2/Nucb2/Pde8b/Per2/Pfkfb2/Pla2g6/Pomc	22	0.852803
BP	GO:0009141	nucleoside triphosphate metabolic process	19/736	0.089419	Adk/Ampd2/Ampd3/Atp5c1/Atp5h/Bid/Cad/Ctps2/Entpd5/Gck/Hk1/Ifng/Igf1/Ndufv2/Nme4/Nudt1/P2rx7/Pfkfb2/Prkag2	19	0.688247
BP	GO:0051881	regulation of mitochondrial membrane potential	8/736	0.089419	Bid/Bok/Dcn/Hsh2d/Mapt/P2rx7/Rnf122/Spg20	8	0
BP	GO:0032368	regulation of lipid transport	10/736	0.098593	Abcb4/Anxa2/Cry2/Nr1h2/Nucb2/P2rx7/Pla2g6/Pltp/Pomc/Prkcd	10	0.632456
BP	GO:0015711	organic anion transport	24/736	0.098735	Abcb1a/Abcb4/Atp11a/Atp8b4/Atp9b/Gja1/Il1rn/Nos2/P2rx7/Per2/Pla2g6/Plin2/Pltp/Prelid3b/Prkcd/Slc13a1/Slc17a4/Slc19a1/Slc27a1/Slc35c2/Slc43a1/Slc5a6/Slco4a1/Stra6l	24	1.632993
MF	GO:0005085	guanyl-nucleotide exchange factor activity	19/728	0.016389	Abr/Arfgef1/Arhgef3/Arhgef39/Cyth2/Dennd4c/Eef1d/Hps1/Iqsec2/Itsn2/Kalrn/Mycbp2/Plekhg2/Plekhg5/Rasgef1a/Rpgr/Spata13/St5/Wdr41	19	-0.229416
KEGG	mmu04710	Circadian rhythm	9/340	0.000309	Arntl/Cry2/Cul1/Npas2/Per1/Per2/Per3/Prkag2/Rorc	9	0.333333
KEGG	mmu04621	NOD-like receptor signaling pathway	23/340	0.001098	Casp8/Ccl12/Defa17/Defa2/Defa21/Defa22/Defa23/Defa24/Defa3/Defa30/Defa31/Defa33/Defa34/Defa36/Defa5/Ifi204/Ifi207/Mfn2/Nlrp3/Nlrx1/Oas2/P2rx7/Prkcd	23	3.127716
KEGG	mmu05202	Transcriptional misregulation in cancer	23/340	0.001241	Arnt2/Ccnd2/Cdkn1b/Defa17/Defa2/Defa21/Defa22/Defa23/Defa24/Defa3/Defa30/Defa31/Defa33/Defa34/Defa36/Defa5/Fcgr1/Gria3/H3f3a/Igf1/Ntrk1/Per2/Zbtb16	23	3.544745
KEGG	mmu05150	Staphylococcus aureus infection	15/340	0.005046	Defa17/Defa2/Defa21/Defa22/Defa23/Defa24/Defa3/Defa30/Defa31/Defa33/Defa34/Defa36/Defa5/Fcgr1/Krt14	15	3.356586
