# Supplementary material for: The Protective Effects of Sour Orange (Citrus aurantium L.) Polymethoxyflavones on Mice Irradiation-Induced Intestinal Injury
Source: Molecules. 2022 Mar 16;27(6):1934. doi: 10.3390/molecules27061934 (PMC8948989; doi:10.3390/molecules27061934)
Supplement: Supplementary file 1 [file molecules-27-01934-s001.zip › molecules-1625294-supplementary/3-Supplemental Materials/Table S2 Standards information.pdf]

Table S2. The flavonoid standards used in this study.

No.	Standard name	Molecular formula	Molecular mass	CAS No.	Purity	Purchase source*
S1	Eriocitrin	C27H32O15	596.5321	13463-28-0	≥ 98.0 %	1
S2	Rutin	C27H32O14	580.5319	14259-46-2	≥ 98.0 %	1
S3	Naringin	C21H22O8	580.5319	10236-47-2	≥ 98.0 %	1
S4	Hesperidin	C28H34O15	610.5628	520-26-3	≥ 98.0 %	1
S5	Diosmin	C28H32O15	608.5433	520-27-4	≥ 98.0 %	1
S6	Didymin	C28H34O14	594.5674	14259-47-3	≥ 98.0 %	1
S7	Neohesperidin	C28H34O15	610.5619	13241-33-3	≥ 95.0 %	2
S8	Hesperetin	C16H14O6	302.2835	520-33-2	≥ 95.0 %	1
S9	Naringenin	C15H12O5	272.2534	480-41-1	≥ 95.0 %	1
S10	Isosinensetin	C20H20O7	372.3686	17290-70-9	≥ 98.0 %	1
S11	Sinensetin	C20H20O7	372.3686	2306-27-6	≥ 98.0 %	2
S12	3′,4′,5,7 - Tetrathoxyflavone	C19H18O6	342.3403	855-97-0	≥ 97.0 %	3
S13	4′,5,6,7 - Tetrathoxyflavone	C19H18O6	342.3403	1168-42-9	≥ 98.0 %	4
S14	Nobiletin	C21H22O8	402.3949	478-01-3	≥ 98.0 %	1
S15	3′,4′,3,5,6,7,8-Hetamethoxyflavone	C22H24O9	432.4355	1178-24-1	> 98.0%	4
S16	4′,5,7-Trimethoxyflavone	C18H16O5	312.3277	5631-70-9	≥ 98.7 %	3
S17	5-Hydroxy-6,7,8,3′,4′-pentamethoxyflavone	C20H20O8	388.3716	2174-59-6	≥ 98.0 %	4
S18	Tangeretin	C20H20O7	372.3686	481-53-8	≥ 98.0 %	1

*1-Chroma Dex; 2- Sigma-Aldrich; 3- Chengdu Chroma-Biotechnology Co., Ltd.; 4- Chengdu Biopurify Phytochemicals Ltd.
