# Supplementary material for: The Protective Effects of Sour Orange (Citrus aurantium L.) Polymethoxyflavones on Mice Irradiation-Induced Intestinal Injury
Source: Molecules. 2022 Mar 16;27(6):1934. doi: 10.3390/molecules27061934 (PMC8948989; doi:10.3390/molecules27061934)
Supplement: Supplementary file 1 [file molecules-27-01934-s001.zip › molecules-1625294-supplementary/3-Supplemental Materials/Table S3 Standard curves of monomers.pdf]

**Table S3.** Standard curves of monomers detected in this study

| No. | Linearity range (μg/mL) | Regression equation | Correlation coefficient ($R^2$) | LOD (μg/mL)* | LOQ (μg/mL)* | Recovery rate (%) | RSD* （%） | Precision (n=6) | |
|---|---|---|---|---|---|---|---|---|---|
| | | | | | | | | Intra-day | Inter-day |
| S1 | 2.0 - 400 | y=1591.1x – 2112.3 | 0.9998 | 0.7167 | 1.7917 | 3.58 | 3.57 | 1.07 | 1.27 |
| S2 | 0.5-100 | y=7010.5x – 2298.3 | 0.9998 | 0.0795 | 0.4773 | 97.22 | 2.37 | 0.79 | 0.81 |
| S3 | 0.4-200 | y=7029x – 5025.6 | 0.9993 | 0.2514 | 0.5148 | 94.03 | 2.91 | 0.82 | 0.89 |
| S4 | 1.5-600 | y=6954.3x + 1083.2 | 0.9999 | 0.0766 | 0.9152 | 99.79 | 5.77 | 0.77 | 1.25 |
| S5 | 0.4-200 | y=2375.2x – 242.9 | 0.9999 | 0.0539 | 0.7329 | 97.35 | 2.49 | 0.56 | 0.78 |
| S6 | 0.15-50 | y=24995x – 3153.2 | 0.9994 | 0.0290 | 0.0871 | 94.57 | 1.48 | 0.81 | 1.69 |
| S7 | 0.5-25 | y=4739.6x-26233 | 0.9979 | 0.0527 | 0.2766 | 95.37 | 4.71 | 0.97 | 0.36 |
| S8 | 1.5-600 | y=7079.5x+34278 | 0.9998 | 0.0787 | 0.9273 | 99.87 | 5.74 | 0.78 | 1.21 |
| S9 | 0.5-200 | y=7183.1x-16791 | 0.9997 | 0.1798 | 0.4132 | 98.79 | 4.39 | 0.79 | 1.03 |
| S10 | 0.25-30 | y=8564x + 628.8 | 0.9996 | 0.0631 | 0.2314 | 99.33 | 3.14 | 0.78 | 1.38 |
| S11 | 0.25-30 | y=10777x + 933.5 | 0.9999 | 0.0369 | 0.1329 | 100.42 | 4.43 | 0.76 | 1.36 |
| S12 | 0.25-30 | y=11289x + 27.6 | 0.9998 | 0.1114 | 0.2227 | 100.67 | 2.90 | 0.65 | 1.26 |
| S13 | 0.25-30 | y=9067.4x – 11468.2 | 0.9994 | 0.1297 | 0.1622 | 103.61 | 3.49 | 0.75 | 0.59 |
| S14 | 0.5-50 | y=9587x + 2886.9 | 0.9992 | 0.0532 | 0.1926 | 102.62 | 3.04 | 0.77 | 1.22 |
| S15 | 0.25-100 | y=8790.4x + 721.7 | 0.9991 | 0.0625 | 0.2249 | 102.46 | 3.66 | 0.75 | 1.67 |
| S16 | 0.25-50 | y=4772.7x – 2342.2 | 0.9992 | 0.0487 | 0.1487 | 103.69 | 2.76 | 0.83 | 0.45 |
| S17 | 0.5-80 | y=6266.9 – 237.8 | 0.9999 | 0.0932 | 0.2496 | 101.12 | 3.51 | 0.70 | 1.13 |

| | | | | | | | | | |
|---|---|---|---|---|---|---|---|---|---|
| S18 | 0.25-50 | y=10893x – 1888.9 | 0.9991 | 0.3180 | 0.9325 | 99.99 | 2.99 | 0.73 | 1.00 |

* LOD, limit of detection; LOQ, Limit of Quantitation; RSD, relative standard deviation.
